# Effect of Qiangji Jianli decoction on mitochondrial respiratory chain activity and expression of mitochondrial fusion and fission proteins in myasthenia gravis rats

**DOI:** 10.1038/s41598-018-26918-z

**Published:** 2018-06-05

**Authors:** Jingwei Song, Xiaowen Lei, Wei Jiao, Yafang Song, Weijing Chen, Jinqiu Li, Zhiwei Chen

**Affiliations:** Institute of Spleen-Stomach, Guangzhou University of Chinese Medicine, Guangzhou, 510006 China

## Abstract

Myasthenia gravis (MG) is an autoimmune neuromuscular disease characterized by the production of antibodies against acetylcholine receptors (AChRs). Qiangji Jianli (QJJL) decoction is an effective traditional Chinese medicine (TCM) that is used to treat MG. Our study aimed to investigate the effect of QJJL decoction on MG and to clarify the mechanism by which QJJL regulates mitochondrial energy metabolism and mitochondrial fusion and fission (MFF). SPF female Lewis rats were administered Rat 97–116 peptides to induce experimental autoimmune myasthenia gravis (EAMG). The treatment groups received QJJL decoction (7.8 g/kg, 15.6 g/kg and 23.4 g/kg). Mitochondria were extracted from gastrocnemius tissue samples to detect respiratory chain complex enzymatic activity. Quantitative PCR and western blot analysis were performed to detect Mfn1/2, Opa1, Drp1 and Fis1 mRNA and protein expression, respectively, in the mitochondria. Transmission electron microscopy examination was performed to show the improvement of mitochondria and myofibrils after QJJL treatment. The results indicated that QJJL decoction may attenuate MG by promoting the enzymatic activity of respiratory chain complexes to improve energy metabolism. Moreover, QJJL decoction increased Mfn1/2, Opa1, Drp1 and Fis1 mRNA and protein expression to exert its curative effect on MFF. Thus, QJJL decoction may be a promising therapy for MG.

## Introduction

Myasthenia gravis (MG) is an autoimmune disease resulting from morphological and functional alterations in the neuromuscular junction (NMJ) and is characterized by fatigable oculobulbar and limb weakness. Some data show that the morbidity of MG is (0.5–5)/100000, the life-time prevalence of MG is 10/100000, and the annual incidence of MG is 2.5/100000. The disease decreases quality of life and may even be life threatening^1^. The current treatments for MG include cholinesterase inhibitors, thymectomy, immunosuppressive agents and short-term immunomodulation via plasma-exchange and intravenous immunoglobulin^2^. However, there is no correlation between antibody titer and disease severity. Moreover, some patients experience severe side effects from their immunosuppressive therapy^3^. These findings indicate that the immune system is not the only system involved in the pathogenesis of MG. Current studies have found that mitochondria play an important role in the pathogenesis of the disease^4^.

Mitochondria are highly dynamic organelles that display continuous movement and continuously undergo fusion and fission to form their reticulums. These complex processes are precisely regulated by many types of mitochondrial fusion and fission (MFF)-related proteins, such as Mfn1/2, Opa1, Drp1 and Fis1^5^. Mitochondria regulate their morphology and function by maintaining the balance between fusion and fission. Disruption of this balance and changes in the expression levels of the above proteins result in several changes. For example, mitochondrial fusion efficiency decreases in Mfn1/2 gene-knockout rats, which results in morphological fragmentation^6,7^. Opa1 plays a main role in stabilizing the mitochondrial cristae. However, homozygous mutations in this gene may lead to cristae breakage, resulting in mitochondrial hypertrophy^8^. Drp1 impacts microtubule distribution to maintain mitochondrial morphology during fission^9^. Additionally, Fis1 and Drp1 are the main mitochondrial fission factors in mammalian cells. Moreover, the Drp1- and Fis1-mediated upregulation of PINK1 expression in senescent human endothelial cells present an additional protection mechanism against oxidative damage in mitochondria^10^. As a Fis1-interacting protein, Caf4 causes Dnm1 to localize in the mitochondrial outer membrane^11^. MFF also influences mitochondrial energy metabolism, and changes in the expression of the above proteins may also have many effects on this process. The absence of Mfn2 adversely affects respiration in fibroblasts, which decreases the mitochondrial membrane potential^12^. However, Mfn2 overexpression increases the mitochondrial membrane potential in HeLa cells^13^. Opa1 not only protects the integrity of the inner membrane to prevent proton leakage but also facilitates efficient electron transport between the respiratory chain complexes inside this membrane^14^. Abnormal MFF results in axonal degeneration and neuromuscular diseases. Moreover, variations in Mfn2 expression may cause energy metabolism abnormalities by slowing down the mitochondrial transport velocity in sensory axons and motor axons^15^.

Professor Deng created Qiangji Jianli (QJJL) decoction according to his clinical experience^16^. QJJL decoction is a traditional Chinese medicine prescription modified from Buzhong Yiqi decoction and includes Radix astragali, Radix codonopsis pilosulae, Atractylodes macrocephala, Radix angelicae sinensis, Cimicifugae rhizoma, Radix bupleuri, Pericarpium citri reticulatae and Radix glycyrrhizae^17^. The active components of Radix bupleuri can attenuate the collapse of the mitochondrial membrane potential and repair DNA damage^18^. Isoliquiritigenin, which is isolated from Radix glycyrrhizae, inhibits ROS generation and has neuroprotective effects^19^. Selenium, which is found in Radix glycyrrhizae, has antagonistic effects on cadmium-induced apoptosis, promotes mitochondrial dynamic balance and maintains mitochondrial energy metabolism^20^. Astragalus polysaccharides protects mitochondria by scavenging ROS, preventing autophagy and ameliorating mitochondrial dysfunction, changes that ultimately improve mitochondrial energy metabolism^21^. Randomized controlled trials of QJJL decoction have demonstrated that the drug has important effects in patients with MG. The decoction has been used clinically for more than 30 years^22,23^. QJJL capsules decrease acetyl choline receptor antibody (AChR-Ab) levels^24^. Professor Wang found that QJJL capsules have superior effects in experimental polymyositis and improve various types of muscle damage, such as muscle fiber denaturalization, putrescence, atrophy, and rupture^25^. However, our understanding of the effects of QJJL decoction at the molecular level is limited. Therefore, it is essential to investigate the effect of QJJL decoction on the pathogenesis of MG.

In this report, we used QJJL decoction to treat experimental autoimmune myasthenia gravis (EAMG) rats and examined mitochondrial respiratory chain complex enzymatic activity. Quantitative PCR and western blot analysis were applied to detect Mfn1/2, Opa1, Drp1 and Fis1 mRNA and protein expression, respectively, in the mitochondria. We aimed to investigate whether QJJL decoction maintains the balance between mitochondrial fusion and mitochondrial fission at a higher frequency to stabilize mitochondrial morphology and thus improve mitochondrial energy metabolism to repair muscle cell damage in MG.

## Results

### Evaluation of the EAMG rat model

**Figure 1:**
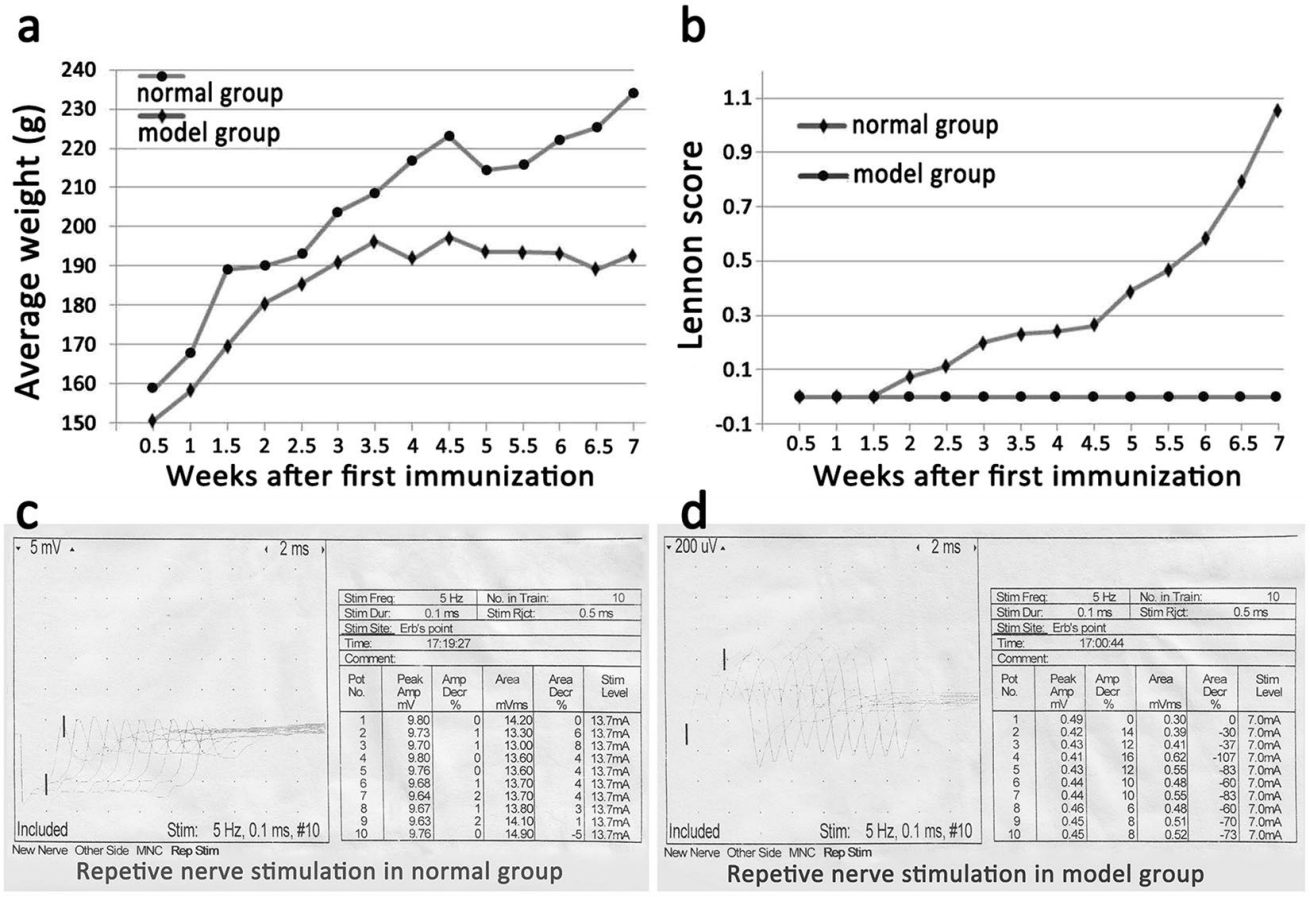
Evaluation of the EAMG rat model. Before immunization, the weight of the normal group rats remained stable, and the rats exhibited sustained growth. The model group rats grew slowly during the week of the first immunization, and their weights tended to be stable and thus did not increase after the second booster immunization (**a**). Before QJJL treatment, 8 rats in the model group died due to severe myasthenia gravis (MG) symptoms, and 54 rats remained alive. During the second week after the first immunization, some of the rats in the model group began to display symptoms of MG and were found to have Lennon scores up to 0.5. With the passage of time, the MG symptoms displayed by the rats in the model group gradually worsened. At 1 week after the last immunization, virtually all of the model group rats had a Lennon score of 0.5 or greater. Specifically, 94.44% of the rats had a score of 0.5 or higher, and 75.93% of the rats had a score of 1.0 or higher. The rats in the blank control group had a score of 0 (**b**). The degree of electrical attenuation was detected by repetitive nerve stimulation (RNS) in both the normal and model groups at one week after the third immunization. The percentage change between the first and fifth action potentials was the attenuation value D, and a percentage change > 10% was considered positive (**c**,**d**).

We used Rat 97~116 peptide as an antigen to immunize Lewis rats to prepare the EAMG rat model. Twenty days after the first immunization, the majority of the model rats began to lose weight and displayed decreased food and water intake compared with the normal rats. Some of the model rats were found to have a Lennon score of 0.5. After two booster immunizations, the model group rats displayed less crying behavior, less activity, more yellow hair and less luster than the control group rats. Furthermore, almost all the model group rats had Lennon scores above 0.5. (Fig. 1a,b). After three separate immunizations, 8 of the 62 rats in the model group had died, and 54 of the rats remained alive. Ultimately, 43 rats were evaluated by electromyography (Fig. 1c,d). The rate at which the EAMG rat model was successfully established was 79.63%.

### Effects of QJJL decoction on EAMG rats

There were no statistical differences in average weight or Lennon scores among the groups before QJJL treatment (Table 1). However, the average weight of the model group significantly decreased after given normal saline orally (P < 0.05). The average weight of the high-, middle- and low-dose groups significantly increased after QJJL decoction treatment (P < 0.05). The average weight of the high-, middle-, or low-dose groups were significantly higher than the model group after QJJL decoction treatment, respectively (P < 0.05) (Table 2).

**Table 1:** Comparison of the average weight and Lennon scores for each group before QJJL decoction treatment (Mean ± SD).

Group	n	Average weight(g)	Lennon score
Model group	8	190.60 ± 8.06^#^	1.25 ± 0.54^*^
High-dose group	8	193.64 ± 5.54^#^	1.15 ± 0.34^*^
Middle-dose group	8	196.36 ± 6.87^#^	0.91 ± 0.49^*^
Low-dose group	8	192.90 ± 9.18^#^	1.14 ± 0.12^*^

^#^P > 0.05, there was no difference in average weight among four groups before QJJL decoction treatment; ^*^P > 0.05, there were no differences in Lennon scores among the four groups before QJJL decoction treatment.

**Table 2:** Comparison of average weight among the groups before and after QJJL decoction treatment.

Group	n	average weight (before QJJL decoction treatment) (g)	average weight (after QJJL decoction treatment) (g)
Normal group	8	235.75 ± 14.98	263.13 ± 9.52
Model group	8	190.60 ± 8.06	182.60 ± 10.91^#^
High-dose group	8	193.64 ± 5.54	215.00 ± 9.94^#*^
Middle-dose group	8	196.36 ± 6.87	215.91 ± 6.74^#*^
Low-dose group	8	192.90 ± 9.18	206.45 ± 5.71^#*^

(Mean ± SD). Comparison of average weight in the model, high-, middle- and low-dose groups before and after QJJL decoction treatment, respectively, ^#^P < 0.05; comparison of average weight between the model group and the high-, middle- and low-dose groups after QJJL decoction treatment, ^*^P < 0.05.

### QJJL decoction improves myofibrils and mitochondria damage in skeletal muscle of EAMG rats

**Figure 2:**
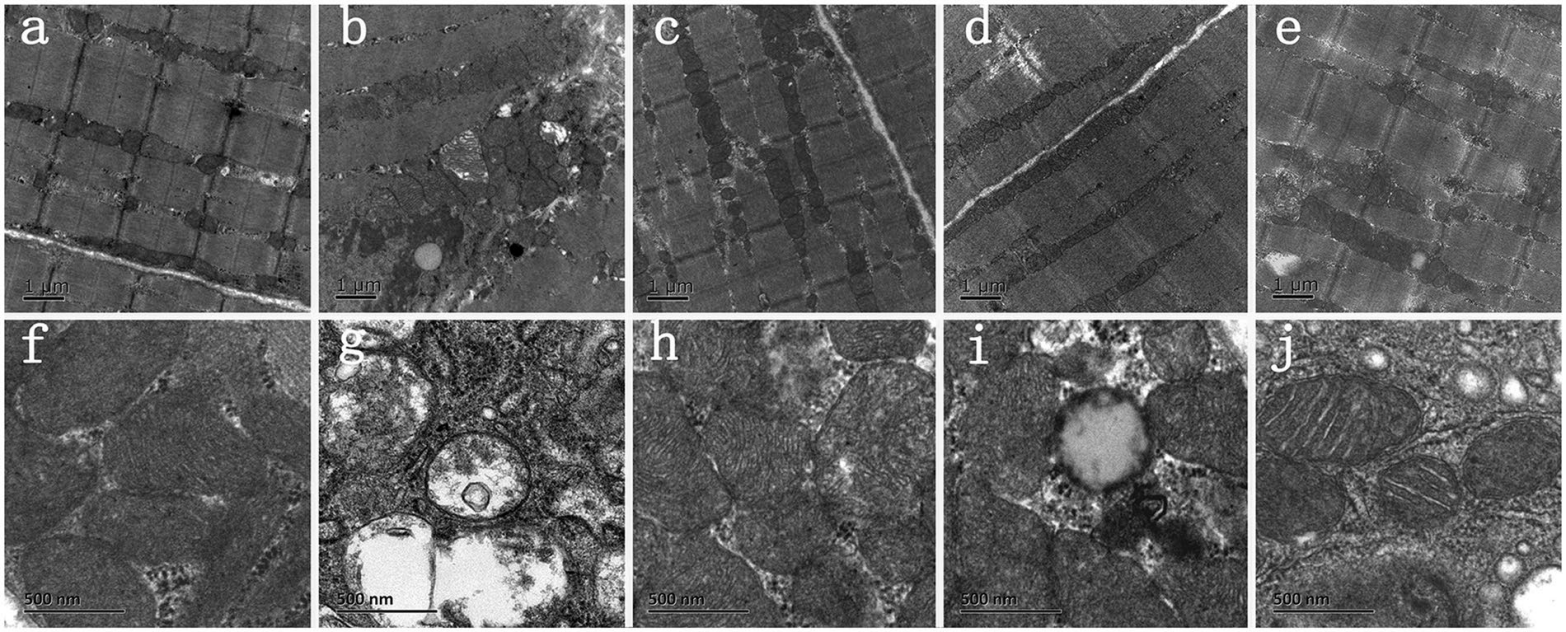
Transmission electron microscopy examination (TEM). Normal mitochondria and myofibrils in skeletal muscle of normal group (**a**,**f**). Transmission electron microscopy examination of the model (**b**,**g**), high- (**c**,**h**), middle- (**d**,**i**), low-dose (**e**,**j**) group.

After 4 weeks of treatment, transmission electron microscopy (TEM) examination was performed. Normal mitochondria and myofibrils in skeletal muscle of normal group (Fig. 2a,f). The myofibrils with alternating dark and light bands and regular Z lines in the middle of I bands (Fig. 2a). Mitochondria were observed between the myofibrils with intact morphology and dense cristae (Fig. 2f). Disordered myofibrils and mitochondria with fuzzy cristae in skeletal muscle of model group (Fig. 2b,g). Mitochondria were observed with ruptured mitochondrial membrane and irregular cristae. Some of them even leaving empty vesicle-like structures (Fig. 2g). The severity of mitochondrial damage decreased gradually with increase in decoction dose (Fig. 2c–e,h–j).

### Effects of QJJL decoction on skeletal muscle mitochondrial respiratory chain complex enzymatic activity in EAMG rats

**Figure 3:**
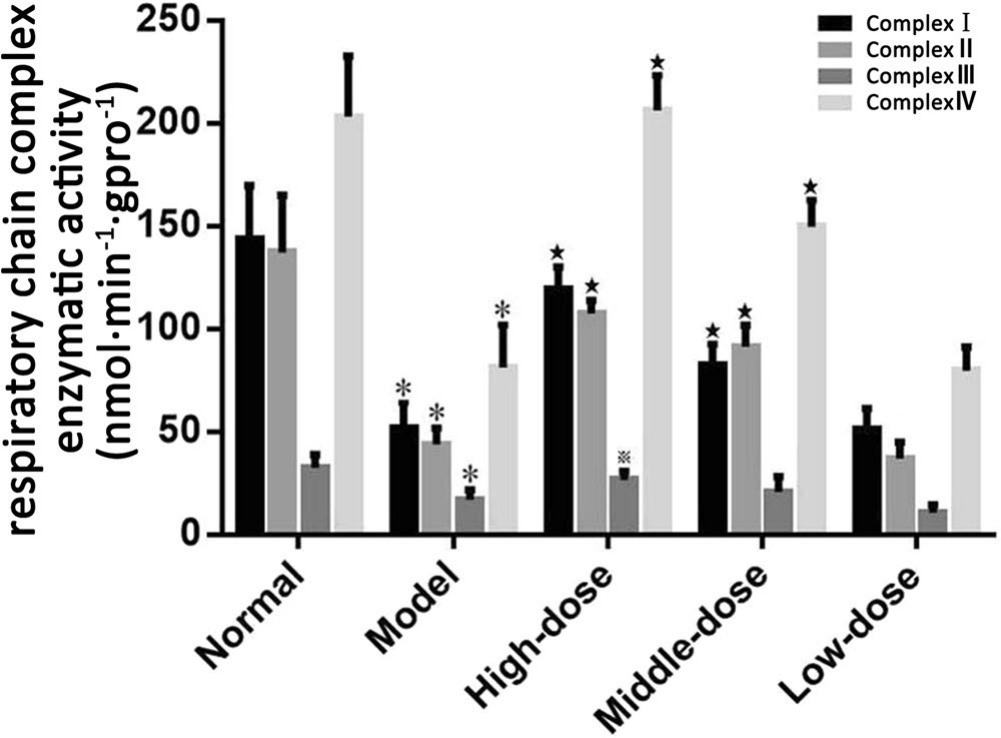
Skeletal muscle mitochondrial respiratory chain complex enzymatic activity in EAMG rats after 4 weeks of treatment (nmol·min^−1^·gpro^−1^, Mean ± SD, n = 8). Compared with normal group, ^*^P < 0.01; compared with model group, ^★^P < 0.01, ^▲^P < 0.05.

After 4 weeks of treatment, the enzymatic activity of skeletal muscle mitochondrial respiratory chain complexes I~IV in the model group was significantly decreased compared with that in the normal group (P < 0.01). The enzymatic activity of skeletal muscle mitochondrial respiratory chain complexes I~IV in the high- and middle-dose groups, especially the enzymatic activity of complexes I, II, and IV, was increased compared with that in the model group (P < 0.01); however, enzymatic activity of complex III was significantly increased only in the high-dose group (P < 0.05). The enzymatic activity of skeletal muscle mitochondrial respiratory chain complexes I~IV in the low-dose group was decreased compared with that in the model group. However, the difference in enzymatic activity between the two groups was not statistically significant (P > 0.05) (Fig. 3, Supplementary Table S1).

### Quantitative analysis of Mfn1, Mfn2, Opa1, Drp1 and Fis1 mRNA expression in the skeletal muscles of the rats in each group after QJJL decoction administration

To understand the effect of QJJL decoction on Mfn1 mRNA expression, we performed quantitative PCR and used the total RNA extracted from the samples as a template to collect data after 4 weeks of treatment. All the experimental data obtained through the PCR amplification experiments were manipulated according to the experimental 2-delta Ct data processing method: ΔCt = mean (gene Ct − internal reference Ct) ± SD; ΔΔCt = mean (sample gene Ct − reference sample Ct) ± SD (if no reference sample was available, the sample which had largest CT value was used as a reference). The starting quantity of the relative sample = mean (2-delta Ct) ± SD.

**Figure 4:**
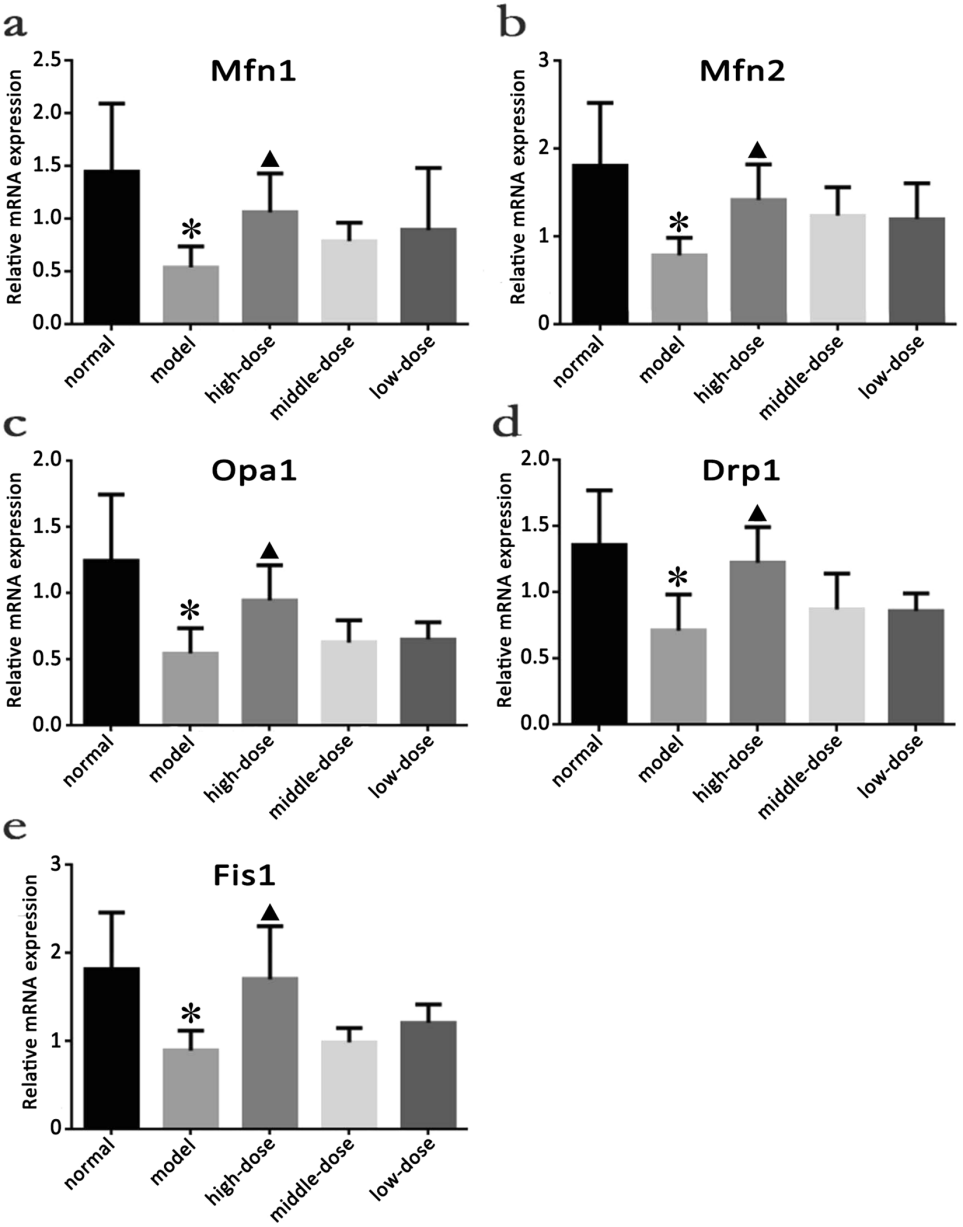
Effect of Qiangji Jianli (QJJL) decoction on Mfn1, Mfn2, Opa1, Drp1 and Fis1 mRNA expression in the skeletal muscle of the EAMG rats in the normal, model, high-dose, middle-dose and low-dose groups. (**a**) Mfn1, (**b**) Mfn2, (**c**) Opa1, (**d**) Drp1, and (**e**) Fis1 mRNA expression after 4 weeks of treatment (Mean ± SD). Compared with normal group, ^*^P < 0.05; compared with model group, ^▲^P < 0.05.

Mfn1, Mfn2, Opa1, Drp1 and Fis1 mRNA expression levels in the model group were significantly decreased compared with those in the normal group (P < 0.05). Mfn1, Mfn2, Opa1, Drp1 and Fis1 mRNA expression levels in the high-dose group were significantly increased compared with those in the model group (P < 0.05), while Mfn1, Mfn2, Opa1, Drp1 and Fis1 expression levels in the middle- and low-dose group tended to higher than those in the model group; however, the differences in the mRNA expression levels of the above proteins between the middle- and low-dose and model groups were not significant (P > 0.05) (Fig. 4, Supplementary Table S2 and S3).

### Effect of QJJL decoction on Mfn1, Mfn2, Opa1, Drp1 and Fis1 protein expression in EAMG rats

**Figure 5:**
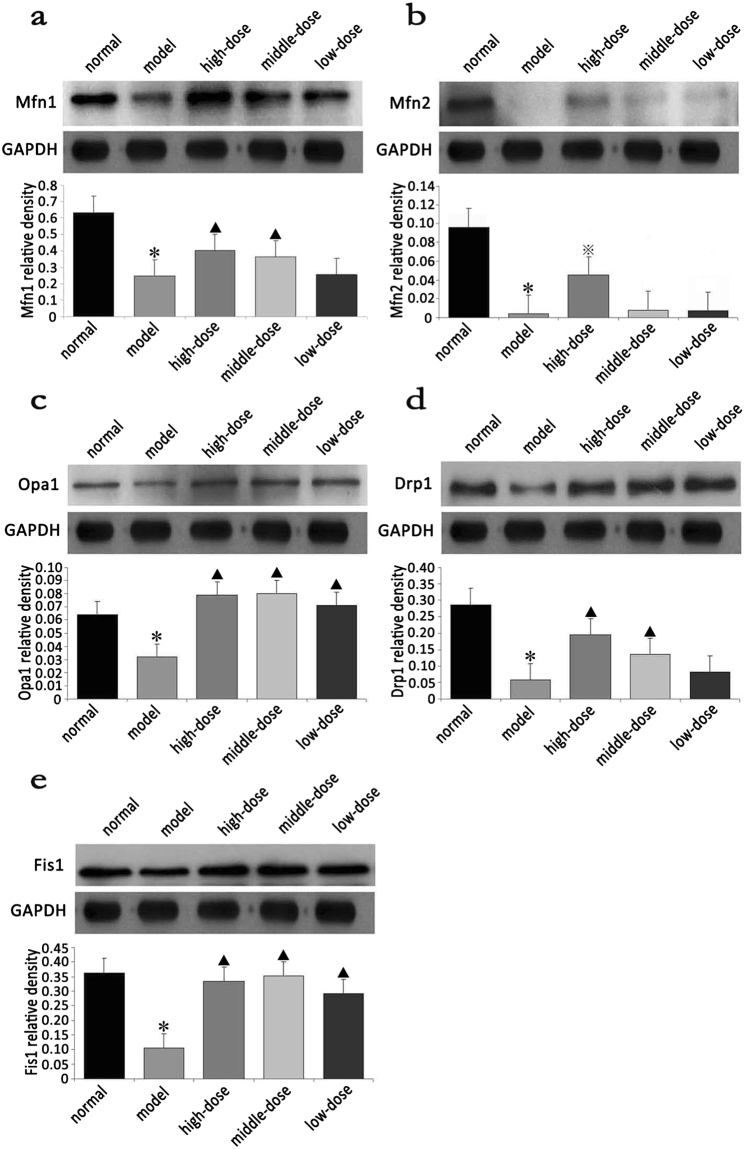
Effect of QJJL decoction on skeletal muscle mitochondrial dynamics of the Lewis rats. Representative Western blot images and quantification of (**a**) Mfn1, (**b**) Mfn2, (**c**) Opa1, (**d**) Drp1 and (**e**) Fis1 protein levels. (Mean ± SD), GAPDH was used as a loading control. Compared with normal group, ^*^P < 0.01; compared with model group, ^▲^P <0.01, ^※^P <0.05.

Before QJJL treatment, Mfn1, Mfn2, Opa1, Drp1 and Fis1 protein expression in the model group was significantly decreased compared with that in the normal group (P < 0.01). After 4 weeks of QJJL treatment, Mfn1, Mfn2, Opa1, Drp1 and Fis1 protein expression in the skeletal muscles of the high-, middle- and low-dose QJJL decoction groups was increased compared with that in the skeletal muscles of model group. Mfn1, Mfn2, Opa1, Drp1, Fis1 protein expression levels in the high-dose group were significantly increased compared with those in the model group (P < 0.05 or P < 0.01). Opa1, Drp1, Mfn1 and Fis1 protein expression levels in the middle-dose group were significantly increased compared with those in the model group (P < 0.01); however, there was no significant difference in Mfn2 expression levels between the middle-dose and model groups (P > 0.05). Opa1 and Fis1 protein expression levels in the low-dose group were significantly different from those in the model group (P < 0.01). (Fig. 5, Supplementary Table S4 and S5).

## Discussion

The EAMG model is an important model for studying the pathogenesis, prevention and treatment of MG. We used immunologic techniques to induce a disease which clinical manifestations, characteristic ultrastructure, electrophysiologic changes, immunologic features, pharmacologic treatments and characteristic histologic changes are similar to MG^26^. We induced immunoreactions by activating AChR-specific T cells with a synthetic AChR α-subunit which known as Rat 97~116 peptide. Patrick *et al*. immunized rabbits with an AChR purified from the electric eel and succeeded in inducing an animal model of EAMG^27^. Although the electric eel electrical organ has an abundance of AChRs, the complex steps required to purify and acquire the AChR restrict the large-scale application of the electric eel model. Passive immunization via the passive transfer AChR monoclonal antibodies is less expensive than other methods, but it is difficult to collect satisfactory serum samples from patients with MG. Thus, we chose to utilize multi-peptide-specific immunization for our study. Different animals have different susceptibilities to MG. We chose a rat model instead of a mouse, rabbit or guinea pig model because rats possess a more potent anti-infection capacity than these animals. Some studies have shown that MG susceptibility in rats ranges from strong to weak in the following order: Wistar > Munich > Fischer > Lewis > Buffalo > Brown > Norway > ACI > Wastar Kyoto > Kopen-hagen > Wastar^28^. Lewis rats are chose as our animal model due to their sensitivity to MG, which facilitates its rapid onset^29^. Some studies found that young female rats were more susceptible to EAMG than male rats^30,31^. Finally, we decided to induce MG by immunizing SPF female Lewis rats aged 6 weeks. Eight of the 62 SPF female Lewis rats died during the experiment. Ultimately, MG was successfully induced in 43 rats, as demonstrated by electromyography. Thus, the model was successfully established in 79.63% of rats, a percentage higher than the 72.2% noted in the study by Baggi *et al*.^32^. We surmised that inducing EAMG with Rat 97–116 peptide may enable us to clarify the pathogenesis of MG and to identify an important target for antigen-specific therapies for MG. Our method is not only reliable but also easy to replicate and convenient to operate.

The respiratory chain of the mitochondrial inner membrane features an enzyme system comprising 4 redox complexes. The mitochondria are responsible for energy metabolism by respiratory chain-mediated oxidative phosphorylation. A study by Wang L *et al*.^33^ suggested that Buzhong Yiqi pills can improve respiratory chain complex II and IV and ATPase activity in the rat brain after acute exercise. Our research found that the enzymatic activity of respiratory chain complexes I-IV in the model group was significantly lower than that in the normal group (P < 0.01), indicating that declining enzymatic activity in EAMG rats may disrupt electron transport in the respiratory chain. These changes may ultimately limit ATP production and slow energy metabolism. After QJJL decoction treatment, enzymatic activity in treatment groups improved markedly compared with that in the model group. In particular, enzymatic activity in respiratory chain complexes I, II and IV in the high- and middle-dose groups increased significantly compared with that in the model group (P < 0.01). The electron micrographs indicated that the ultrastructure of mitochondria and myofibrils in skeletal muscle had improved after QJJL decoction treatment. These results demonstrated that QJJL decoction may enhance enzymatic activity, restore the mitochondrial respiratory chain and improve energy metabolism in the skeletal muscle of EAMG rats. Enzymatic activity in the low-dose group was slightly decreased compared with that in the model group, but the difference in enzymatic activity between the two groups don’t has statistical significance (P > 0.05). Perhaps the low-dose decoction can not reach the effective therapy.

The mammalian mitofusins Mfn1 and Mfn2 are present in three distinct molecular complexes, in which they promote mitochondrial fusion and maintain mitochondrial function^6^. OPA1 is a dynamic mitochondrial inner membrane GTPase whose pleckstrin homology domain interacts with various phospholipids to target proteins and coordinate the tubulation of the outer and inner membranes^34^. Opa1 then further closes to the cristae. S-Opa1 cooperates with L-Opa1 to interact with Mfn1/2 to promote mitochondrial fusion^35^. Drp1 overexpression promotes cytochrome C release and mitochondrial fission^36^. However, dominant-negative Drp1 mutant (Drp1K38A) overexpression inhibits mitochondrial fragmentation and caspase activation to slow down apoptosis^37^. Drpl down-regulation reduces mitochondrial membrane potential and ATP generation^38^. Mitochondrial quality control mechanisms maintain mitochondrial function by selective fusion, fission and mitochondrial autophagy (mitophagy), so as to isolate and remove dysfunctional mitochondria, respectively^39^. The study of Mouli *et al*. shows that mitochondrial functional components can redistribute unevenly during fusion events, which causes parent mitochondria to generate two dissimilar daughter mitochondria by asymmetric fission. One daughter mitochondria will be preferentially removed due to its lower membrane potential and indication of OXPHOS impairment. This mechanism causes the cell to segregate and remove damaged mitochondrial material at a much faster rate^40^. The mathematical model of the mitochondrial quality control process also shows that a higher mitochondrial fusion-fission frequency allows faster clearance of mutant mtDNA^41^. Enhanced MFF-related proteins expression can restore mitochondrial dynamics after hydrogen peroxide-induced impairment^42^. The above experimental observations reveal that mitochondria may maintain their morphology and biological function by balancing fusion and fission at a higher frequency. Mfn1/2, Opa1, Drp1 and Fis1 mRNA and proteins expression were significantly decreased in EAMG rats compared with normal rats (P < 0.01). These results demonstrated that MG is closely related to MFF and that MG may suppress MFF by down-regulating the expression of MFF-related mRNA and proteins. These changes may result in less mitochondria and abnormal morphology, as well as reductions in membrane potential or ATP generation, leading to biological functional disruptions. Due to energy metabolism abnormalities, the rats with MG displayed limb weakness, withered hair and weight loss. Mfn1/2, Opa1, Drp1 and Fis1 mRNA and protein expression levels were significantly increased in the high-dose group compared with the model group (P < 0.05). Moreover, Mfn1/2, Opa1, Drp1 and Fis1 protein expression levels increased in a dose-dependent manner after treatment with QJJL. In detail, Mfn1/2 and Drp1 protein expression levels in the low-dose group were higher than the model group, but there was no statistical significance (P > 0.05). Mfn1, Opa1, Drp1 and Fis1 protein expression levels in the middle- and high-dose groups increased significantly compared with that in the model group (P < 0.01). Based on the above studies regarding their effects on mitochondrial quality control mechanisms, these results indicated that QJJL decoction may upregulate the expression of MFF-related mRNA and proteins to elevate the frequency of MFF events, and thus segregate and remove damaged mitochondrial material at a much faster rate to maintain mitochondria morphology and their biological function.

Different from previous studies, which focused much attention on the immune system, our study focused on MFF in skeletal muscle cells damage and attempted to clarify its involvement in the pathogenesis of MG. This is the first study to examine the restorative effect of QJJL decoction on mitochondrial energy metabolism and to investigate the effect of QJJL decoction on Mfn1/2, Opa1, Drp1 and Fis1 mRNA and protein expression. Our study aimed to identify potential therapeutic targets for MG in Chinese medicine. Future studies directed at determining the exact molecular signaling mechanisms underlying MG development, particularly *in vitro* studies regarding the factors that control mitochondrial biosynthesis and energy metabolism, are warranted.

## Methods

### Chemicals and reagents

Rat 97–116 peptide (DGDFAIVKFTKVLLDYTGHI) was purchased from GL Biochem Co., Ltd. (Shanghai, China). Chloral hydrate (B/N: 20150715) was purchased from Damao Chemical Reagent Factory (Tianjin, China). Phosphate buffer solution (PBS, pH 7.4, Lot: 8115207) was purchased from Gibco BRL Co., Ltd. (USA). Complete Freund’s adjuvant (CFA, P/N: P5506) and incomplete Freund’s adjuvant (IFA, P/N: P5881) were purchased from Sigma-Aldrich Shanghai Trading Co., Ltd. (Shanghai, China). Neostigmine and atropine sulfate were provided by the Department of Pharmacy of the First Affiliated Hospital of Guangzhou University of TCM. The mitochondrial respiratory chain complex kits (I~IV, P/N: FHTA-2-Y, FHTB-2-Y, FHTC-2-Y, and FHTD-2-Y) used herein were purchased from Comin Biotechnology Co., Ltd. (Suzhou, China). Chloroform was purchased from the Chemical Reagent Factory (Guangzhou, China). Isopropanol was purchased from Xilong Chemical Co., Ltd. (Guangdong, China). Anti-Mfn1 antibodies (P/N: sc-50330), anti-Mfn2 antibodies (P/N: sc-50331), anti-Drp1 antibodies (P/N: sc-32898) and anti-Fis1 antibodies (P/N: sc-98900) were purchased from Santa Cruz. Anti-Opa1 antibodies (P/N: ab 157457) were purchased from Abcam. Goat anti-rabbit secondary antibodies (P/N: BA1054, NO. BST11E06A54) and goat anti-mouse secondary antibodies (P/N: BA1050, NO. BST10D02A) were purchased from Boster Biological Technology Co., Ltd. (WuHan, China). GAPDH mouse monoclonal antibodies (P/N: KM9002) were purchased from Sanjian (Hanzhou, China). All other chemicals used in the study were standard commercial products of analytical reagent grade.

### Experimental animals

The study protocol was approved by the Institutional Animal Care and Use Committee of Guangzhou University of Chinese Medicine and all experiments were performed in accordance with relevant guidelines and regulations. Seventy-two SPF female Lewis rats (6 weeks of age, 129.72 ± 8.39 g) were provided by Vital River Laboratory Animal Technology Co, Ltd. (Beijing, China). The experimental animal certification number was SCXK (Jin) 2012–0001.

### Rat model preparation and experimental groups

We used an experimental method described previously to induce EAMG in Lewis rats^43,44^. After one week of adaptive feeding, 72 Lewis rats were randomly divided into groups A (n = 10) and B (n = 62). The rats in group B were immunized with Rat 97–116 peptides (100 μg/200 μl) in CFA via the hind-foot pads, waist and abdomen. They were boosted with Rat 97–116 peptides (50 μg/200 μl) in IFA on days 30 and 45. The rats in group A were given an equivalent dose of PBS. Finally, 8 rats from group A were randomly assigned to a normal group. Thirty-two rats from group B were divided into 4 groups containing 8 rats per group. These 4 groups were designated as the model group, high-dose herb group, middle-dose herb group, and low-dose herb group, respectively.

### Herbal preparation and animal treatment

Qiangji Jianli (QJJL) decoction is a TCM prescription modified from the Buzhong Yiqi decoction and includes Radix astragali, Radix codonopsis pilosulae, Atractylodes macrocephala, Radix angelicae sinensis, Cimicifugae rhizoma, Radix bupleuri, Pericarpium citri reticulatae and Radix glycyrrhizae at the following compatibility ratio: 20:6:5:4:3:3:1:1. All herbs were purchased from a Xinyuanchun pharmacy (Guangzhou, China) and were identified by the College of Chinese Pharmaceutical Science (Guangzhou University of TCM, Guangzhou, China). The medicinal herbs were decocted for 45 min, after which they were soaked for 30 min following the addition of a 10-fold larger volume of water. Then the herbs were filtered out and the liquid was collected and marked as D1. The filtered herbs were re-suspended in a 6-fold volume of water and re-decocted for 30 min. Then the herbs were filtered out again and the liquid was collected and marked as D2. Finally, the resulting decoction, which was marked as D2, was mixed with D1 in a water-bath until the concentration needed for the high-dose experimental group was obtained. The decoction was then stored in a refrigerator at 4 °C for later use. After herb preparation, the decoction was administered to the animals. All rats received treatment by gavage once a week for 4 weeks.

The treatment protocol was as follows:

The normal and model groups were given saline.

The high-dose herb group was given QJJL decoction (23.4 g/kg•d).

The middle-dose herb group was given QJJL decoction (15.6 g/kg•d).

The low-dose herb group was given QJJL decoction (7.8 g/kg•d).

In addition, each rat was injected with the same volume of fluid (1 ml/100 g•d).

### Observation indexes and evaluation of the rat model

Each group was observed, and data pertaining to the following parameters were recorded daily: movement, food intake, stool character, hair color and luster. Animal weights were measured and recorded 2 times a week. MG severity was classified as follows, according to the Lennon test^45^: Grade 0, normal strength; Grade 1, mildly reduced movement and a weak grip; Grade 2, exhaustion and weakness at rest; Grade 3, moribund; and Grade 4, dead. Grades of 0.5, 1.5 or 2.5 were used when the syndromes were of severities between specific grades. The results are shown as the mean. We used the study by Deng *et al*.^46^ as a reference. The successful establishment of MG was confirmed by intraperitoneal injections of neostigmine (37.5 μg/kg), along with atropine sulfate (15 μg/kg). The rats in the PBS and model groups experienced improvements in their muscle strength after 12 min, effects that persisted for 2–12 hours if MG was induced successfully.

### Diagnostic value of repetitive nerve stimulation (RNS)

Model group rats were fastened to an apparatus after the intraperitoneal injection of 10% chloral hydrate (350 mg/kg). We then inserted a stimulating electrode into the gastrocnemius muscle, near the sciatic nerve, and a reference electrode into the abdominal subcutaneous tissue. One recording electrode was inserted in the subcutaneous tissue near the Achilles tendon, and another was inserted into the medial head of the gastrocnemius muscle. Finally, all the electrodes received a 5 Hz electrical stimulus. The stimulus was performed 10 times, and the nerve response was recorded. The percentage change between the fifth and first responses was taken as the attenuation value D. The result was considered positive when the percentage decrease between the first and fifth responses was greater than 10%.

### Transmission electron microscopy (TEM)

After 4 weeks of treatment, each gastrocnemius muscle fraction of 1 mm × 1 mm × 1 mm was fixed in 2.5% glutaraldehyde for 2 hours at 4 °C. After six washes in phosphate buffer solution, the tissues were postfixed with 1% osmium tetroxide on ice for 2 hours and washed three times with phosphate buffer solution. Then the tissues were embedded in Epon 812 after dehydration in grade ethanol. After sectioned with an ultramicrotome (UC-7, Shanghai Leica Microsystems Co., Ltd, China), the tissue was stained with uranyl acetate and lead citrate. Finally, ultrathin sections were examined with a transmission electron microscope (JEM-1400, JEOL Ltd, Tokyo, Japan).

### Assay of the activity of mitochondrial respiratory chain complexes I~IV in skeletal muscle cells in EAMG rats

After undergoing treatment for 4 weeks, the five groups were fasted for 24 h and then killed to obtain fresh gastrocnemius tissue samples. First, 200 mg of gastrocnemius tissue was cut into pieces and placed in a precooling centrifuge tube after being washed with physiological saline. Second, the tissue homogenate was placed into another precooling tube and centrifuged at 1000 × g for 3 min at 4 °C after being ground in 1.5 ml of precooling cell lysate. Third, the supernatant was collected, and 0.5 ml of supernatant was mixed with 0.5 ml of medium buffer in a microtube. The samples were eventually centrifuged at 15000 × g for 10 min at 4 °C, which resulted in the mitochondria precipitating at the bottom of centrifuge tube. The precipitate was then mixed with 0. 2 ml of wash buffer and centrifuged at 15000 × g for 10 min at 4 °C, after which the precipitate was collected and mixed with 75 µl of store buffer to complete the mitochondrial exaction procedure. Before detection, the mitochondrial respiratory chain complexes were subjected to freezing and thawing at −20 °C and 37 °C, respectively, for 3 cycles. All activities of complexes I-IV were detected on a ultraviolet visible spectrophotometer (UVmini-1240, Shimadzu factory). Enzyme activity was expressed as nmol·min^−1^·gpro^−1^. Assay of complex I (NADH-CoQ reductase, EC 1.6.5.3) activity was calculated by measuring the oxidation rate of NADH at 340 nm. Assay of complex II (Succinate Dehydrogenase, EC 1.3.99.1) activity was evaluated by measuring the reduction of dichloroindophenol (DCIP) at 605 nm. Assay of complex III (CoQ-cytochrome c reductase, EC 1.10.2.2) activity was calculated by measuring the increase of reduced cytochrome C at 550 nm. Assay of complex IV (cytochrome c oxidase, EC 1.9.3.1) activity was determined by measuring the reduction of reduced cytochrome C at 550 nm.

### PCR for analysis of Mfn1/2, Opa1, Drp1 and Fis1 mRNA expression

Skeletal muscle tissue specimens were ground in liquid nitrogen and treated with 1 ml of Trizol solution before being refrigerated at −80 °C and then incubated for 5 min at room temperature. A total of 0.2 ml of chloroform was added to the mixture, which was vortexed for 15 s. After incubating for 5 min at room temperature, the mixture was centrifuged at 12000 rpm for 15 min at 4 °C, and the resulting supernatant, which contained RNA, was transferred to a new microtube. After the addition of isopropanol (0.6 ml), the solution was gently agitated and then incubated for 1 hour at −20 °C. The samples were subsequently centrifuged at 12000 rpm for 15 min at 4 °C, after which the supernatant was discarded, and ethanol (75%, 1 ml) was added to the mixture to purify the nucleic acid. The mixture was then centrifuged at 12000 rpm for 15 min at 4 °C to remove the residual supernatant. The dried pellet was dissolved in 20 μl of sterile deionized water, and the samples were analyzed by spectrophotometry. Sample purity and concentrations were quantified using an ultraviolet spectrophotometer (K5500, Beijing Kaiao Technology Development Co., Ltd). The 260/280 “nucleic acid absorbance/protein absorbance” and 260/230 “nucleic acid absorbance/organic absorbance” ratios were determined, after which the samples were subjected to electrophoresis at a constant voltage of 100 V for 20 min. The results showed that the quality of the exacted RNA was acceptable and that the RNA was thus appropriate for use in subsequent experiments. The mRNA sequences of the target genes were downloaded from the GenBank database. Specific primers were designed with CDS Region, and GAPDH (293 T cell, 1 μg of total RNA) was used as the housekeeping gene to test the efficiency of the procedure. Primer express 2.0 software was used to design the primers and probe. The sequences of the primers are as follows:

Sequence name: R-Mfn1

Forward primer: 5′-ATCTGGTGGAGATACAGGGCT-3′

Reverse primer: 5′-TCCCACAGCATTGCGTTGAT-3′

Sequence name: R-Mfn2

Forward primer: 5′-GCTCAGTCGGTTGGAAGTCA-3′

Reverse primer: 5′-GAAAGGAGTGCCTGCCTGAT-3′

Sequence name: Opa1

Forward primer: 5′-GGCACTTCAAGGTCGTCTCA-3′

Reverse primer: 5′-CACTGCTCTTGGGTCCGATT-3′

Sequence name: Dnm1l (Drp1)

Forward primer: 5′-AGGTTGCCCGTGACAAATGA-3′

Reverse primer: 5′-CACAGGCATCAGCAAAGTCG-3′

Sequence name: Fis1

Forward primer: 5′-ACGCCTGCCGTTACTTCTTC-3′

Reverse primer: 5′-GCAACCCTGCAATCCTTCAC-3′

### Quantitative PCR

(1). We used RNA from the above samples to construct a reaction system comprising the following reactants: 3.6 μl of RNase-free water, 0.2 μl of forward primer (C) (10 μM), 0.2 μl of reverse primer, 5 μl of SYBR Green SuperMix and 1 μl of cDNA template. (2). All samples were retrotranscripted in the 3′-5′ direction using a thermocycler and the following program, which was repeated for 40 cycles: 95 °C for 20 s, 95 °C for 10 s, 60 °C for 30 s and 70 °C for 1 s. (3). Immediately after amplification, melt curve analysis was performed at 95 °C for 10 s, and stepwise annealing was performed at temperatures increasing from 70 °C to 95 °C in increments of 0.5 °C.

### Western blot analysis of Mfn1/2, Opa1, Drp1 and Fis1 protein expression

After being prepared with RIPA lysis buffer and phenylmethylsulfonyl fluoride (PMSF), the gastrocnemius muscle cell lysates were centrifuged at 12000 rpm for 5 min at 4 °C. The supernatant was then collected, and the protein concentration was determined via the bicinchoninic acid (BCA) method. The extracted cellular proteins were subjected to SDS-PAGE, transferred to polyvinylidene difluoride (PVDF) membranes, and then blocked with 5% nonfat milk in TBST for 1 hour. The blots were subsequently probed with anti-Opa1 antibodies (diluted 1:1000, rabbit monoclonal antibody) and several rabbit polyclonal antibodies, including anti-Mfn1 antibodies, anti-Mfn2 antibodies, anti-Drp1 antibodies and anti-Fis1 antibodies (all diluted 1:500), overnight at 4 °C. The samples were then incubated with goat anti-rabbit secondary antibodies (diluted 1:5000) for 50 min before being washed in TBST 5 times. The samples were then treated with ECL to identify the immunoreactive bands. Densitometry analysis of the immunoreactive bands was performed using Gelpro (version 32) software.

### Statistical analysis

All statistical data were analyzed by SPSS 19.0 software. The results are expressed as the mean ± SD. Statistical comparisons between two groups would be evaluated with Student’s unpaired t-test. Normally distributed data were analyzed by one way ANOVA followed by Least-significant Difference (LSD) test. In addition, heteroscedasticity was interpreted with Dunnett T3 analysis. Statistical significance was set at P < 0.05 or P < 0.01.

## Electronic supplementary material

Supplementary Information
