# Supplementary material for: Effect of Qiangji Jianli decoction on mitochondrial respiratory chain activity and expression of mitochondrial fusion and fission proteins in myasthenia gravis rats
Source: Sci Rep. 2018 Jun 5;8:8623. doi: 10.1038/s41598-018-26918-z (PMC5988663; doi:10.1038/s41598-018-26918-z)
Supplement: Supplementary file 1 — Supplementary Information [file 41598_2018_26918_MOESM1_ESM.pdf]

Effect of Qiangji Jianli decoction on mitochondrial respiratory chain activity and expression of mitochondrial fusion and fission proteins in myasthenia gravis rats

Jingwei Song[1,+], Xiaowen Lei[1,+], Wei Jiao[1], Yafang Song[1,*], Weijing Chen[1], Jinqiu Li[1], Zhiwei Chen[1]

[1] Institute of Spleen-Stomach, Guangzhou University of Chinese Medicine, Guangzhou, 510006, China.
[*] corresponding author, email: sallysongyue@163.com
[+]these authors contributed equally to this work

Group	Complex Ⅰ	Complex Ⅱ	Complex Ⅲ	Complex Ⅳ
Normal group	143.72±25.88	137.15±27.71	32.40±6.44	203.32±29.37
Model group	51.56±13.01*	43.79±7.98*	16.90±4.91*	81.05±21.13*
High dose group	119.08±11.03★	107.49±6.43★	26.51±4.29※	206.06±17.38★
Middle dose group	82.13±10.69★	91.39±10.36★	20.77±7.49	149.74±13.05★
Low dose group	51.10±10.32	37.01±7.89	10.54±3.92	79.68±11.38

Table S1. Skeletal muscle mitochondrial respiratory chain complex enzymatic activity in EAMG rats ($nmol \cdot min^{-1} \cdot gpro^{-1}$, n=8, Mean±SD). Compared with normal group, *P<0.01; compared with model group, ★P<0.01, ※P<0.05.

Group	cases	Mfn1	Mfn2	Opa1
Normal group	8	1.45±0.64	1.78±0.72	1.24±0.50
Model group	8	0.54±0.20*	0.79±0.20*	0.55±0.19*
High dose group	8	1.06±0.37▲	1.41±0.41▲	0.94±0.27▲
Middle dose group	8	0.79±0.18	1.23±0.32	0.63±0.17
Low dose group	8	0.89±0.59	1.20±0.41	0.65±0.13

Table S2. Effect of Qiangji Jianli (QJJL) decoction on Mfn1, Mfn2 and Opa1 mRNA expression in the skeletal muscle of the EAMG rats (n=8, mean±SD). Compared with normal group, *P＜0.05；Compared with model group，▲P＜0.05.

Group	cases	Drp1	Fis1
Normal group	8	1.36±0.42	1.81±0.64
Model group	8	0.71±0.27*	0.89±0.22*
High dose group	8	1.22±0.27▲	1.70±0.60▲
Middle dose group	8	0.87±0.27	0.98±0.16
Low dose group	8	0.85±0.14	1.21±0.21

Table S3. Effect of Qiangji Jianli (QJJL) decoction on Drp1 and Fis1 mRNA expression in the skeletal muscle of the EAMG rats (n=8, mean±SD). Compared with normal group, *P<0.05; compared with model group, ▲P<0.05.

Group	cases	Mfn1	Mfn2	Opa1
Normal group	8	0.632±0.037	0.096±0.003	0.064±0.006
Model group	8	0.245±0.017*	0.004±0.002*	0.032±0.006*
High dose group	8	0.403±0.017▲	0.045±0.006※	0.079±0.007▲
Middle dose group	8	0.361±0.010▲	0.008±0.001	0.080±0.010▲
Low dose group	8	0.255±0.021	0.007±0.001	0.071±0.010▲

Table S4. Mfn1, Mfn2 and Opa1 protein expression levels in the skeletal muscles of the rats in the normal, model, high-dose, middle-dose and low-dose groups (n=8, mean±SD). Compared with normal group, *P＜0.01; compared with model group, ▲P＜0.01, ※P＜0.05.

Group	cases	Drp1	Fis1
Normal group	8	0.286±0.032	0.363±0.020
Model group	8	0.057±0.007*	0.105±0.009*
High dose group	8	0.195±0.021▲	0.333±0.023▲
Middle dose group	8	0.135±0.009▲	0.351±0.021▲
Low dose group	8	0.082±0.007	0.290±0.019▲

Table S5. Drp1 and Fis1 protein expression levels in the skeletal muscles of the rats in the normal, model, high-dose, middle-dose and low-dose groups (n=8, mean±SD). Compared with normal group, *P＜0.01; compared with model group, ▲P＜0.01.
